# The use of Polygenic Scores in a family design of First Episode Psychosis

**DOI:** 10.1192/j.eurpsy.2023.1312

**Published:** 2023-07-19

**Authors:** N. Murillo-Garcia, S. Papiol, S. Barrio-Martínez, M. Sevilla-Ramos, R. Magdaleno-Herrero, Á. Yorca-Ruiz, V. Ortíz-García de la Foz, M. Miguel-Corredera, M. Fatjó-Vilas, R. Ayesa-Arriola

**Affiliations:** 1 Research Institute Marqués de Valdecilla (IDIVAL), Santander, Spain; 2 University Hospital, LMU Munich, Munich, Germany; 3 FIDMAG Sisters Hospitallers Research Foundation, Barcelona; 4 CIBERSAM, Madrid, Spain

## Abstract

**Introduction:**

A wide variety of traits is heritable and has genetic loading, including schizophrenia spectrum disorders (SSDs) and its associated neurocognitive features. The genetic architecture of SSDs is polygenic, with the contribution of thousands of single nucleotide polymorphisms of small effect with an estimated SNP-heritability of 24%. The same occurs with neurocognitive phenotypes such as intelligence or educational attainment. Therefore, the method of polygenic risk scores (PRS) is useful in estimating the genetic burden of such traits. Moreover, the use of PRS in a sample of genetically related individuals would allow analyzing the contribution of genetic and environmental factors involved in the development of the disorder and its candidate endophenotypes.

**Objectives:**

To estimate PRS for schizophrenia, and polygenic scores for intelligence and educational attainment in patients with First Episode Psychosis (FEP), their first-degree relatives (siblings and parents), and a group of healthy controls.

**Methods:**

The sample is comprised of 579 participants of the PAFIP-FAMILIAS project in Santander, Spain (133 FEP patients, their 244 first-degree relatives, and 202 healthy controls). All provided sociodemographic information and completed the same neuropsychological battery. Participants’ DNA was extracted from venous blood samples, and genotyping was performed at the Centro Nacional de Investigaciones Oncológicas (CeGen) by the Global Screening Array v.3.0 panel (Illumina). Data quality control, imputation, calculation of PRS, and genetic association analysis are being performed using PLINK, SHAPEIT, IMPUTE2, SPSS and R.

**Results:**

Data analysis is currently in progress, at the quality analysis stage, in collaboration with the Institute of Psychiatric Phenomics and Genomics (IPPG) in Munich, Germany. We expect to find higher PRS for schizophrenia in FEP patients, while their first-degree relatives will potentially show intermediate risk scores between patients and healthy controls. A similar finding is expected regarding intelligence and educational attainment, as FEP patients may show more genetic burden for low intelligence and education.

**Conclusions:**

The estimation of PRS has demonstrated to be valuable in studying complex traits such as schizophrenia. We believe that by applying this method in a family design can provide interesting insights on the development of SSDs and its potential endophenotypes, and potentially useful in their prevention.

**Disclosure of Interest:**

None Declared

